# Comparative Outcomes of Surgical Stabilization and Nonoperative Management for Rib Fractures: A Quasi-experimental Study

**DOI:** 10.7759/cureus.112322

**Published:** 2026-07-09

**Authors:** Abhishek Kumar, Majid Anwer, Anurag Kumar, Anil Kumar, Rekha Kumari, Sanjay Kumar, Saket Prakash, Deepak Kumar, Deepak Kumar, Jeeshan Khan

**Affiliations:** 1 Trauma Surgery and Critical Care, All India Institute of Medical Sciences, Patna, IND; 2 Anesthesiology (Trauma), All India Institute of Medical Sciences, Patna, IND; 3 Orthopedics, All India Institute of Medical Sciences, New Delhi, IND; 4 General Surgery, All India Institute of Medical Sciences, Patna, IND

**Keywords:** flail chest, inflammatory markers, pic score, rib fixation, rib fractures

## Abstract

Background: Rib fractures are among the most common injuries in blunt thoracic trauma and are associated with significant morbidity due to respiratory compromise, pain, and pulmonary complications. While nonoperative management remains the traditional approach, surgical stabilization of rib fractures (SSRF) has gained attention for improving outcomes in patients with multiple displaced rib fractures and flail chest. This study aimed to compare the clinical and functional outcomes of SSRF vs. nonoperative management in patients with multiple rib fractures at a Level I Trauma Center in Eastern India.

Methods: A quasi-experimental study was conducted from January 2023 to December 2024, including 58 patients aged 18-70 years with multiple displaced rib fractures or flail chest. Patients were divided into two groups: Group A (n = 29) underwent surgical stabilization, and Group B (n = 29) received nonoperative management. Demographic variables, clinical outcomes, duration of hospital stay, ICU stay, ventilatory days, pain scores (Visual Analog Scale), functional outcomes, inflammatory markers, and complications were recorded. Statistical analysis was performed using the Statistical Package for the Social Sciences version 25 (IBM Corp., Armonk, NY), with p < 0.05 considered statistically significant.

Results: Both groups were comparable in demographic characteristics, injury profile, and associated thoracic injuries. Patients in the SSRF group demonstrated significantly shorter hospital stay (9.21 ± 1.99 vs. 12.10 ± 1.52 days, p = 0.01), ICU stay (2.72 ± 0.88 vs. 4.24 ± 0.83 days, p = 0.01), and ventilatory duration (3.03 ± 1.15 vs. 5.38 ± 1.24 days, p = 0.01). Postoperative pain scores were significantly lower in the operative group at all follow-up intervals (p = 0.01). Functional recovery and quality-of-life scores showed progressive improvement in the surgical group. Chronic chest pain was significantly lower in the operative group (1 (3.4%) vs. 26 (89.7%), p = 0.01), with no mortality reported in either group.

Conclusion: SSRF significantly improves clinical outcomes by reducing hospital stay, ICU stay, ventilatory duration, pain severity, and chronic complications compared to nonoperative management. Despite limitations such as a nonrandomized design and a modest sample size, SSRF appears to be an effective treatment strategy for selected patients with multiple rib fractures and flail chest. Further randomized studies with larger sample sizes are recommended to validate these findings.

## Introduction

Rib fractures are among the most frequently encountered injuries in blunt thoracic trauma. According to Ekpe and Eyo [1], they occur in more than half of patients with chest injuries. Pines et al. [2] emphasized that the clinical significance of rib fractures extends beyond direct damage to the bony thorax, as they are often associated with intrathoracic and intra-abdominal organ injuries, compromised respiratory mechanics, and pain-induced hypoventilation. These factors substantially increase the risk of complications such as atelectasis, pneumonia, and respiratory failure.

Haase and Shaikh [3] described the thoracic anatomy, noting that the rib cage consists of 12 pairs of ribs. Rib fractures are typically classified based on their anatomical location (upper, middle, or lower ribs), number of ribs involved, laterality, and fracture pattern (simple, comminuted, displaced, or segmental). Fractures of the upper ribs (first to third) are relatively rare due to protection from the clavicle, scapula, and surrounding muscles; when they do occur, they often indicate high-energy trauma and may be associated with injuries to major vessels such as the subclavian artery and vein, the brachial plexus, and the upper lung lobes. Middle rib fractures (fourth to ninth) are the most common and are strongly associated with pulmonary contusions and pneumothorax. Lower rib fractures (10th-12th) may indicate concomitant injury to abdominal organs such as the liver, spleen, or kidneys, particularly when fractures are posterior and displaced.

Pharaon et al. [4] described flail chest as a severe form of rib injury characterized by multiple consecutive rib fractures, resulting in a free-floating segment of the chest wall. This condition causes paradoxical chest movement during respiration, leading to impaired ventilation, severe pain, and an increased risk of respiratory failure.

Traditionally, rib fractures have been managed conservatively, with an emphasis on adequate pain control, respiratory support, pulmonary hygiene, and early mobilization. Nonoperative management remains the standard approach for most cases. However, Assi and Nazal [5] reported that in certain patient populations, particularly those with flail chest or multiple displaced fractures, conservative treatment may be insufficient, resulting in prolonged ventilator dependence, persistent pain, chest wall deformity, and increased morbidity and mortality.

Kane et al. [6] highlighted that advancements in surgical stabilization of rib fractures (SSRF) have significantly improved the management of complex rib injuries, particularly in critically ill patients. SSRF involves open reduction and internal fixation of fractured ribs using devices such as titanium plates, locking screws, or bioabsorbable materials to restore chest wall stability. Bae et al. [7] identified key indications for SSRF, including flail chest, multiple displaced rib fractures, nonunion or symptomatic malunion, chest wall instability or deformity, and failure of conservative management. Ongoing research and consensus efforts are essential to further refine surgical indications, standardize techniques, and improve patient outcomes in rib fracture management.

Although many international studies have shown positive results with SSRF, there is a lack of evidence from Indian trauma centers, particularly regarding comparative outcomes in day-to-day clinical practice. Findings from Western healthcare systems may not be applicable to the Indian setting, given differences in trauma epidemiology, healthcare infrastructure, patient socioeconomic status, and access to specialized surgical services. In particular, Eastern India has a unique healthcare scenario with a huge burden of road traffic injuries, and financial constraints and variations in trauma care resources may influence patient selection and management strategies. In addition, limited access to surgical stabilization and variation in institutional practices prevent the development of standardized management pathways. Therefore, it is important to generate region-specific evidence to understand the effectiveness and feasibility. Furthermore, evidence from resource-constrained settings remains limited. Therefore, the objective of the present study was to compare SSRF with nonoperative management in patients with multiple displaced rib fractures or flail chest. Specifically, we aimed to evaluate differences in clinical outcomes, including hospital stay, ICU stay, ventilatory support duration, pain reduction, respiratory function, inflammatory markers.

## Materials and methods

We conducted a quasi-experimental study because randomization was not possible in our setting due to the high cost of the surgery. Sample size estimation was based on the primary endpoint of hospital stay duration between treatment arms. Assuming a pooled standard deviation of 2.8 units, a 95% confidence level (α = 0.05), 80% power, and a 1:1 allocation ratio, the calculated sample size was 29 patients per group, for a total of 58 patients. Group 1 (study group) comprised patients who underwent SSRFs. Group 2 (study group) comprised patients who underwent nonoperative management for fractured ribs. Treatment groups were assigned based on institutional SSRF eligibility criteria, surgeon assessment, and patient willingness/financial feasibility for operative intervention. In our setting, random allocation was not possible.

Data collection was done between January 2023 and December 2024. The study received approval from the Ethics Committee of the All India Institute of Medical Sciences, Patna, Bihar.

Participants

All the patients who arrived in our Emergency Department with diagnostic criteria of multiple displaced rib fractures and flail chest, who were between the ages of 18 and 70 years, and who wanted to participate in the study were included. The exclusion criteria were as follows: 1) patients with polytrauma, such as severe traumatic brain injury, severe spinal cord injury, or cardiac injury, and 2) patients with undisplaced rib fractures, those unwilling to participate in the study, and patients with open or infected wounds. Patients were randomized into treatment groups based on clinical assessment, SSRF eligibility based on institutional criteria, surgeon decision-making, and patient willingness/financial feasibility for operative intervention. Patients who had surgical indications and agreed to surgery were subjected to SSRF (Group A), and those who were managed conservatively or unable/unwilling to undergo surgery were included in the group of nonoperative management (Group B). Therefore, the distribution of rib fractures between groups was determined by clinical presentation and treatment pathway rather than random allocation.

Randomization was not possible due to cost restrictions. However, baseline demographic and injury data were compared between the groups to assess comparability. There were no statistically significant differences between the surgical and nonsurgical groups in terms of age, mechanism of injury, pulmonary contusion, hemothorax, pneumothorax, and comorbidities (Tables 1, 2).

**Table 1:** Demographic profile, mode of injury, sites, and presence of pulmonary contusion in both the groups Values are expressed as the mean for continuous variables and n (%) for categorical variables SSRF: surgical stabilization of rib fracture; NOM: nonoperative management; RTA: road traffic accident

Parameter	SSRF (n = 29)	NOM (n = 29)	p value
Age, mean (years)	39 years	40 years	0.80
Sex (male), n (%)	25 (86.2)	22 (75.9)	0.31
Mode of injury, n (%)			
RTA	23 (79)	26 (90)	0.55
Fall from height	4 (13.8)	2 (6.9)	
Assault	2 (6.9)	1 (3.4)	
Site, n (%)			
Right	16 (55.2)	16 (55.2)	0.34
Left	11 (37.9)	13 (44.8)	
B/L	2 (6.9)	0	
Pulmonary contusion, n (%)			
Present	17 (58.6)	13 (44.8)	0.29
Absent	12 (41.4)	16 (55.2)	

**Table 2:** Presence of pneumothorax, hemithorax, and comorbidities in both the groups Values are expressed as n (%) SSRF: surgical stabilization of rib fracture; NOM: nonoperative management

Variables	SSRF (n = 29)	NOM (n = 29)	p value
Hemothorax, n (%)			
Minimal	19 (65.5)	15 (51.7)	0.28
Moderate	10 (34.5)	14 (48.3)	
Pneumothorax, n (%)			
Minimal	16 (55.2)	20 (69.0)	0.27
Moderate	13 (44.8)	09 (31.0)	
Co-morbidities, n (%)	13 (44.8)	09 (31.0)	0.74

Procedure

Phase 1: Initial Assessment

The process begins with the identification of rib fractures. Initial evaluation includes obtaining a contrast-enhanced chest CT scan, assessing respiratory function, and initiating a multimodal pain management protocol, including locoregional analgesia such as nerve blocks.

Phase 2: Identification of Contraindications

Before considering surgical intervention, patients are evaluated for high-risk conditions. In the presence of factors such as shock or ongoing resuscitation, severe traumatic brain injury, acute myocardial infarction, or fractures outside the range of ribs 3-10 (relative contraindications), the patient is managed nonoperatively.

Phase 3: Indications for Surgery

If the patient is stable and none of the above contraindications are present, the next step is to assess for surgical indications. The presence of chest wall instability warrants surgical management.

Ventilator Status

In patients who are on ventilatory support, surgical intervention is considered if there is difficulty in weaning, specifically attributable to rib fractures. In those not requiring ventilator support, surgery is considered when there are three or more rib fractures with at least 50% displacement, along with two or more pulmonary derangements. If these criteria are not fulfilled, the patient is managed conservatively.

Phase 4: Final Clearance and Timing

For patients who meet the criteria for surgery, the final evaluation includes assessment for other higher priority or life-threatening injuries, which must be managed first before proceeding. Once the patient is cleared, the timing of surgery is considered, with optimal intervention ideally performed within 72 hours of injury. After satisfying these conditions, the patient undergoes SSRF.

Surgical procedure

**Figure 1:**
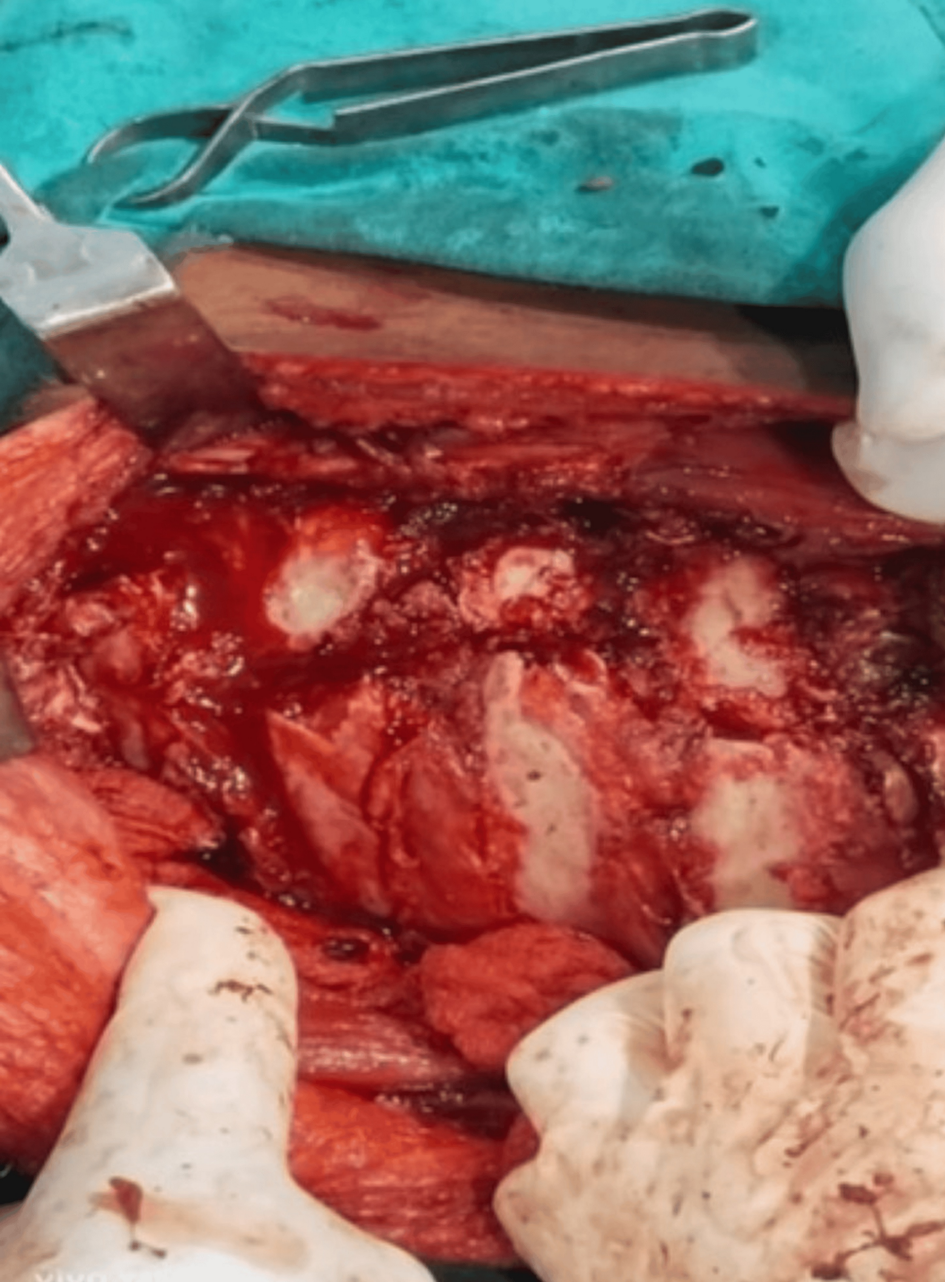
Intraoperative picture showing fractured rib

**Figure 2:**
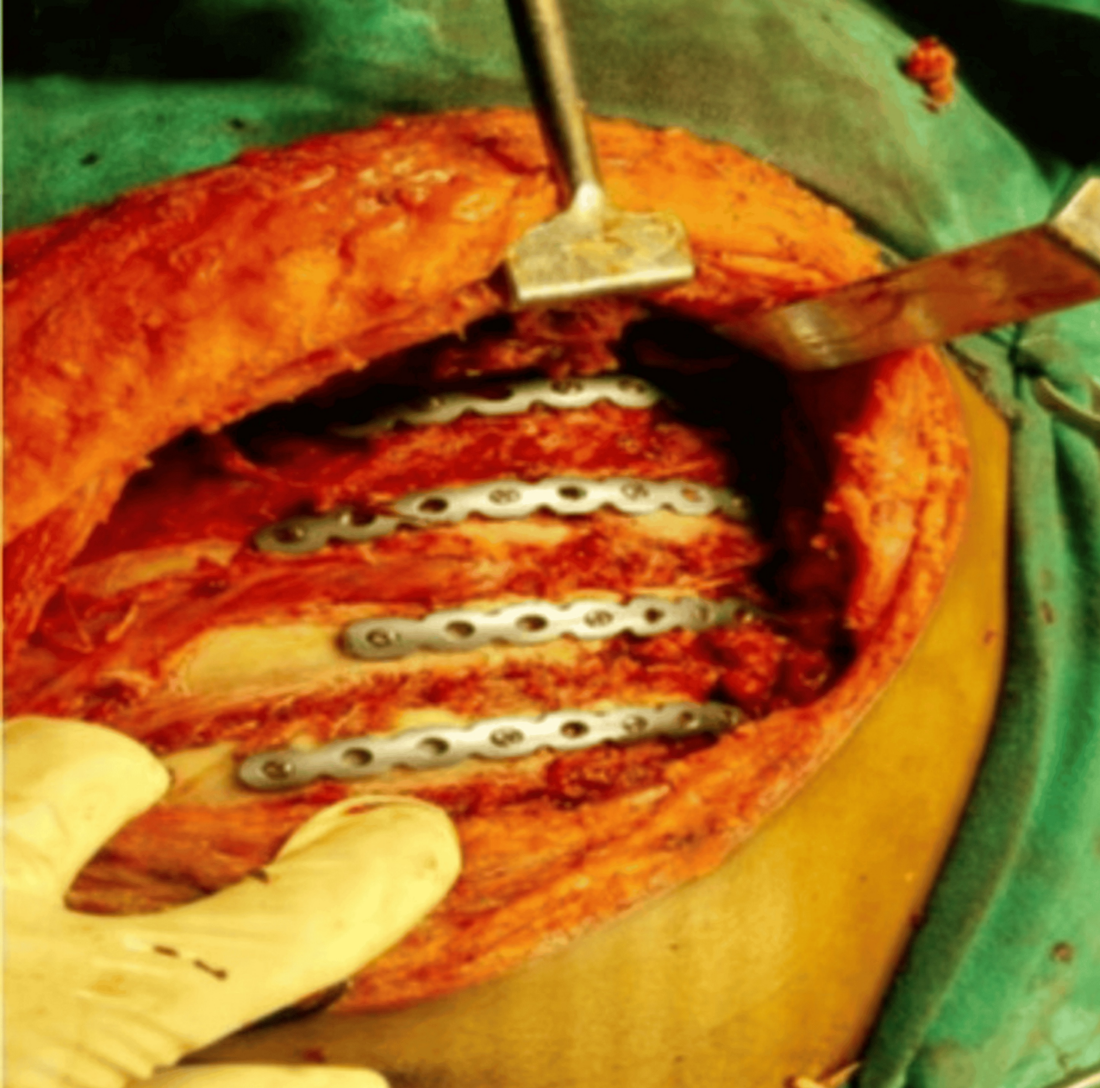
Intraoperative picture showing fixation of rib fracture with screw and locking plate

Surgical management of rib fractures involves open reduction and internal fixation of the affected ribs. Common indications include flail chest, multiple rib fractures, chronic chest pain secondary to rib injury, and chest wall deformity or defects. Preoperative optimization of the patient is essential for successful operative management. Under general anesthesia, an incision is made over the chest wall according to the fractured rib. Fractured rib is exposed, periosteum is elevated, and alignment of the rib is done and fixed with an anatomical plate and locking screw, as shown in Figures 1, 2.

Stabilization was done with locking screw titanium anatomical rib fixation plates. The number of ribs mended was based on fracture pattern, displacement, and chest wall instability established intraoperatively. Whenever practicable, surgical stabilization was preferred to be undertaken within 72 hours of damage. Thoracoscopic assistance was selectively used when clinically necessary, and chest tube insertion was done as per related pleural injuries and intraoperative findings.

Nonoperative management

**Figure 3:**
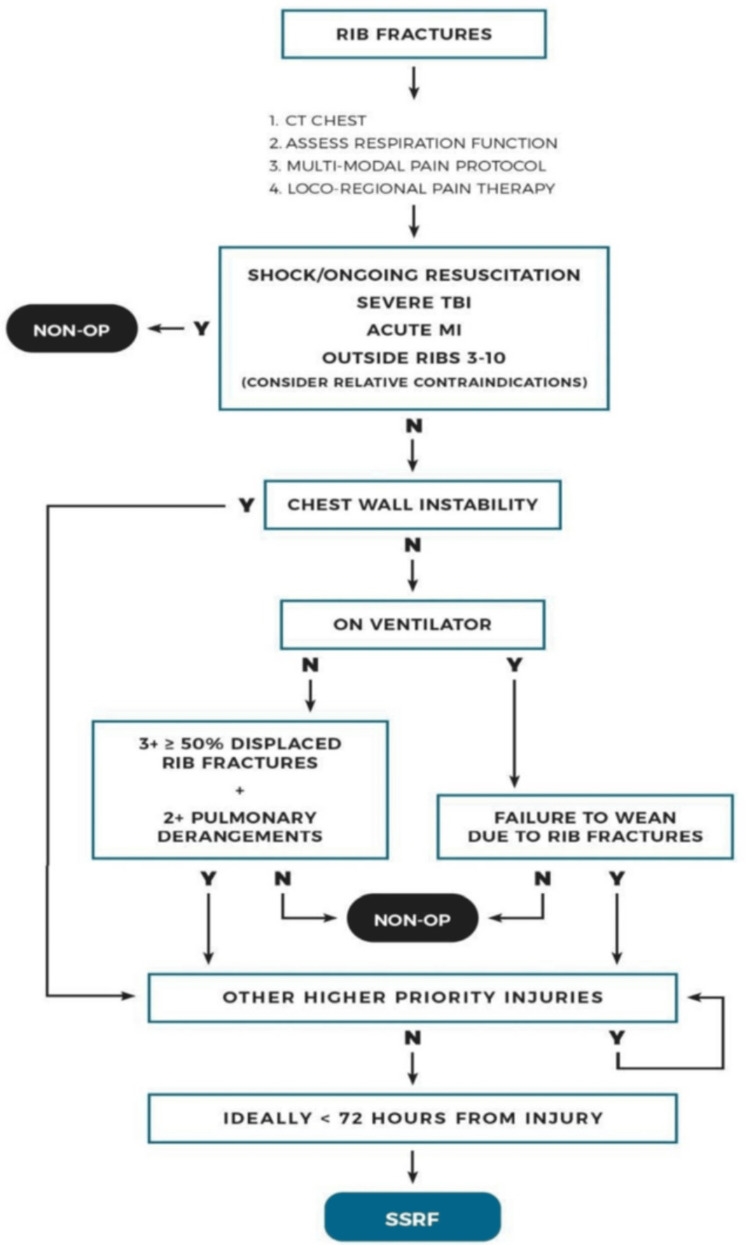
SSRF algorithm SSRF: surgical stabilization of rib fracture; TBI: traumatic brain injury; MI: myocardial infarction

In cases of isolated rib fractures, conservative management is generally sufficient. This typically includes adequate pain control, rest, and the application of ice. Patients should also be encouraged to use an incentive spirometer to reduce the risk of pulmonary atelectasis and prevent splinting. Key components of nonoperative management include aggressive analgesia (systemic opioids, nonsteroidal anti-inflammatory drugs, and regional techniques such as epidurals, paravertebral, or intercostal nerve blocks), incentive spirometry, chest physiotherapy, and, when necessary, oxygen supplementation or mechanical ventilation. The goal is to maintain adequate ventilation, minimize the risk of pneumonia and atelectasis, and promote early ambulation. Figure 3 shows the algorithm for rib fracture management that we followed during the study, as explained above.

## Results

At our center, 58 patients were included in two groups of 29 each. Group A was the operative management group, and Group B was the nonoperative management group.

Data collection method, including settings and periodicity

The data were entered and cleaned using Microsoft Excel (Microsoft Corporation, Redmond, WA) and analyzed statistically using Statistical Package for the Social Sciences, version 25 (IBM Corp., Armonk, NY). Quantitative variables were expressed as the mean ± standard deviation or the median ± interquartile range. Qualitative data were expressed as percentages (%) or proportions. Appropriate statistical tests were used to infer an association between two variables, and a p value of <0.05 was considered statistically significant: 1) chi-square test or Fisher’s exact test was used for categorical variables, and 2) independent samples t-test, paired samples t-test, and repeated measures ANOVA were used for quantitative variables.

Patients' demographics in each group

Table 1 shows that, out of 29 patients in Group A, 10 were in the 18-30 years age group and another 10 were in the 31-45 years age group. Seven patients were in the 46-60 years age group, and two were>60 years, so the mean age of the patients in Group A was 39 years.

In Group B, 14 patients were in the age group of 31-45 years, followed by seven patients in the age group of 18-30 years. Four patients were in the age group of 46-60 years, and four more patients were in the age group of >60 years, so the mean age of patients in Group B was 40 years. The difference between the two groups was not statistically significant (p = 0.44).

Table 1 shows that in both groups, road traffic injury was the most common mode of injury (23 patients in Group A and 26 patients in Group B). Other modes of injury included falls from height and assault in our study. The difference between the two groups was not statistically significant (p = 0.55). The comparison of treatment groups was based on findings of pulmonary contusion on CT. It was present in 17 patients in Group A compared with 13 patients in Group B. However, the difference between the two treatment groups was not statistically significant (p = 0.29).

Table 2 shows a comparison between the two groups for the presence of pneumothorax or hemothorax, which was not significant. Both groups (Groups A and B) had 13 and nine patients with comorbidities, which was not significant.

Table 3 compares the distribution of rib fractures and baseline injury characteristics between the groups. No statistically significant differences were observed in fracture burden, laterality, or associated thoracic injuries, suggesting reasonable baseline comparability between the operative and nonoperative cohorts. So both groups were comparable in terms of demography, mode of injury for the patient, presence of hemothorax or pneumothorax, and presence of comorbidities.

**Table 3:** Baseline rib fracture and injury characteristics between treatment groups Values are given as mean ± SD or n (%). The chi-square and Fisher exact tests were used for categorical variables and the independent t-test for continuous variables SD: standard deviation; ICU: intensive care unit

Variable	Group A	Group B	p value
Mean number of fractured ribs, mean ± SD	7.8 ± 2.1	7.3 ± 2.4	0.41
3-5 fractured ribs, n (%)	8 (27.6)	10 (34.5)	0.57
≥6 fractured ribs, n (%)	21 (72.4)	19 (65.5)	-
Right-sided rib fractures, n (%)	13 (44.8)	15 (51.7)	0.79
Left-sided rib fractures, n (%)	11 (37.9)	10 (34.5)	-
Bilateral rib fractures, n (%)	5 (17.3)	4 (13.8)	-
Multiple displaced rib fractures/flail chest, n (%)	29 (100)	29 (100)	-

Table 4 compares treatment groups by the duration of hospital stay, ICU stay, and ventilatory days. The mean duration of hospital stay among patients in Group B who were managed nonoperatively was 12.10 ± 1.52 days, which was higher than the mean duration of hospital stay among patients in Group A who were managed operatively (9.21 ± 1.99 days). The difference between the two groups was statistically significant (p = 0.01).

**Table 4:** Comparison of treatment groups based on duration of hospital stay, ICU stay, and duration of ventilatory days Independent Student's t-test used assuming equal sample size (n = 29 per group) ^*^Statistically significant (p < 0.05) SD: standard deviation; VAS: Visual Analog Scale

Parameter	Group A (mean ± SD)	Group B (mean ± SD)	p value^*^
Duration of hospital stays (in days)	9.21 ± 1.99	12.10 ± 1.52	0.01
Duration of ICU stay (in days)	2.72 ± 0.88	4.24 ± 0.83	0.01
Duration of ventilatory (in days)	3.03 ± 1.15	5.38 ± 1.24	0.01

The mean duration of ICU stay in Group B patients was 4.24 ± 0.83 days, which was significantly higher than the mean duration of ICU stay in Group A patients (2.72 ± 0.88 days). The difference between the two groups was statistically significant (p = 0.01).

The mean duration of ventilatory days among patients in Group B was 5.38 ± 1.24 days, which was higher than the mean duration of ventilatory days among patients in Group A (3.03 ± 1.15 days). The difference between the two groups was statistically significant (p = 0.01).

Table 5 compares treatment groups based on Visual Analog Scale (VAS) scores at different time intervals. The mean VAS score at baseline was higher in Group A (7.97 ± 1.05) than the mean VAS score in Group B (7.83 ± 1.04). However, the difference between the two groups was not statistically significant (p = 0.62).

**Table 5:** Comparison of treatment groups based on VAS scores at different time intervals Independent Student's t-test used assuming equal sample size (n = 29 per group) SD: standard deviation; VAS: Visual Analog Scale

VAS score	Group A (mean ± SD)	Group B (mean ± SD)	p value
Baseline	7.97 ± 1.05	7.83 ± 1.04	0.62
Postoperatively	3.83 ± 0.66	6.41 ± 0.82	0.01
At one-week follow-up	3.48 ± 0.69	5.34 ± 0.48	0.01
At one-month follow-up	2.41 ± 0.57	4.86 ± 0.87	0.01
At six-month follow-up	0.14 ± 0.51	2.62 ± 0.49	0.01

**Figure 4:**
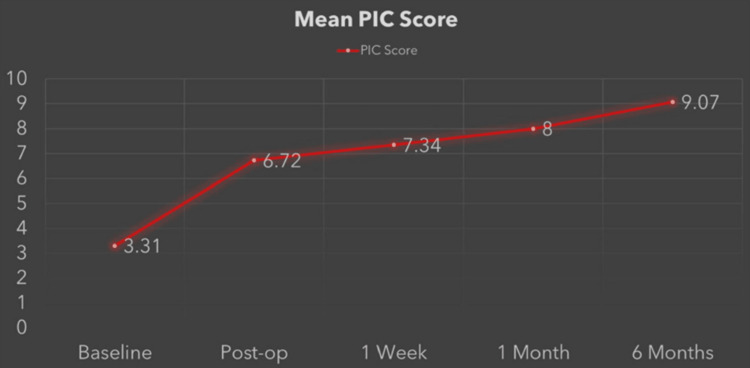
Comparison of PIC score at different time intervals among patients managed operatively PIC: Pain, Inspiratory effort, Cough

At all other time intervals, such as postoperatively and at one-week, one-month, and six-month follow-ups, the difference between the two groups was statistically significant (p = 0.01), with the mean VAS score lower in Group A than in Group B. Figure 4 compares the Pain, Inspiratory effort, Cough (PIC) score at different time intervals among patients managed operatively [8]. The mean PIC score showed an increasing trend, and it increased from 3.31 ± 0.54 at baseline to 9.07 ± 0.26 at six-month follow-up. The difference between PIC scores at different time intervals was statistically significant (p = 0.01).

Table 6 compares different serum inflammatory markers at different time levels among patients managed operatively. All three markers (C-reactive protein (CRP), erythrocyte sedimentation rate (ESR), and total leucocyte count (TLC)) showed a declining trend from baseline to six-month follow-up, and the difference between them was statistically significant (p = 0.01).

**Table 6:** Comparison of different serum inflammatory markers at different time levels among patients managed operatively Repeated measures ANOVA was used CRP: C-reactive protein; SD: standard deviation; ESR: erythrocyte sedimentation rate; TLC: total leucocyte count

Variable	Baseline (mean ± SD)	Postoperatively (mean ± SD)	1-week follow-up (mean ± SD)	1-month follow-up (mean ± SD	6-month follow-up (mean ± SD)	p value
CRP (mg/dL)	116.03 ± 17.47	73.69 ± 8.94	48.79 ± 7.75	22.17 ± 4.75	9.86 ± 3.40	0.01
ESR (mm in the first hour)	45.28 ± 8.68	26.45 ± 4.17	16.52 ± 1.66	11.90 ± 2.27	5.03 ± 1.95	0.01
TLC (per mm^3^)	23.55 ± 3.55	11.11 ± 2.77	8.00 ± 0.84	6.95 ± 0.76	6.27 ± 0.70	0.01

**Figure 5:**
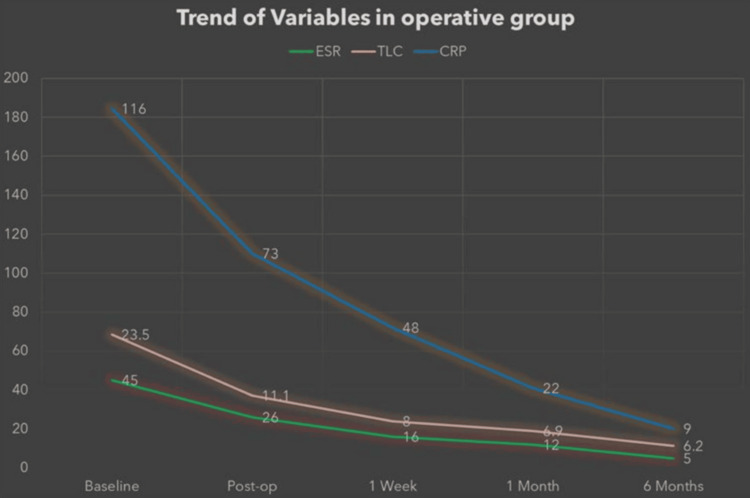
Constant decrease in inflammatory markers until six months in the operated group CRP: C-reactive protein; ESR: erythrocyte sedimentation rate; TLC: total leucocyte count

Figure 5 shows a constant decrease in all three serum inflammatory markers (ESR, TLC, and CRP) and achieving a normal level in six months in postoperative patients.

Both groups were compared on the basis of persistent chronic pain and 30-day mortality. No mortality occurred in either group. However, chronic pain was significantly lower in the operative group. Table 7 shows 30-day mortality and chronic pain in both groups.

**Table 7:** Thirty-day mortality and chronic pain in both the groups

Parameter	Group A, n (%)	Group B, n (%)	p value
Chronic chest pain	01 (3.4)	26 (89.7); χ² ≈ 43.6	0.01
Mortality within 30 days	0	0	-

**Figure 6:**
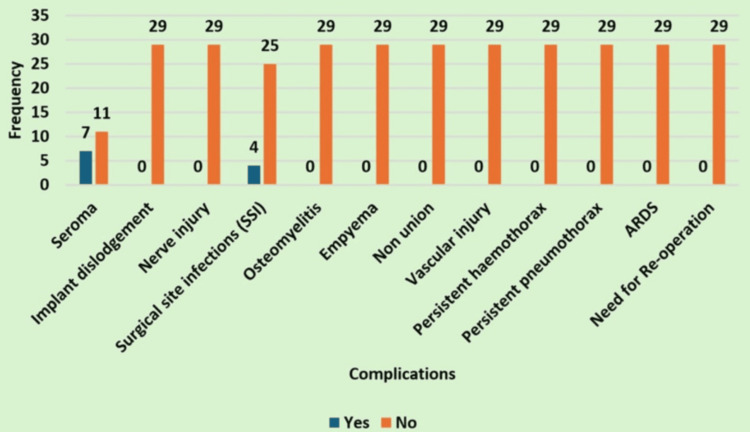
Complications among operated patients ARDS: acute respiratory distress syndrome

Figure 6 shows the distribution of patients managed operatively on the basis of complications. Seroma was seen in seven patients, while surgical site infections were observed in four patients. No other complications, such as implant dislodgement, nerve or vascular injury, osteomyelitis, empyema, nonunion, persistent hemothorax or pneumothorax, acute respiratory distress syndrome, and need for re-operation, were seen in any of the patients in our study.

## Discussion

Fokin et al. [9] found that multiple rib fractures are one of the most common injuries encountered in trauma centers and are associated with approximately 40% of blunt chest injuries. Rib fractures were historically treated nonoperatively after management of head injury, abdominal trauma, or other injuries. However, He et al. [10] described complications and mortality rates, which have been shown to rise with an increase in the patients’ age and the number of fractured ribs. Otaka et al. [11] found that patients with multiple rib fractures of 3 or more and chest abbreviated injury scores ≥3 have significantly higher in-hospital mortality than those who do not, regardless of concomitant injuries. A landmark study by Liu et al. [12] and several meta-analyses have confirmed these benefits, particularly in patients with flail chest and those with more than three rib fractures.

Our study was undertaken with the objective of evaluating and comparing the clinical, functional, and quality-of-life outcomes in patients undergoing surgical vs. nonsurgical treatment for rib fractures. Selection bias cannot be ruled out, as treatment allocation was based on clinical assessment, surgeon judgment, and financial feasibility rather than on randomization. Baseline characteristics were similar, but residual confounding may have affected treatment assignment and outcomes.

In the present study, we found that operative management had a shorter duration of hospital stay than patients who were managed nonoperatively (9.21 ± 1.99 days vs. 12.10 ± 1.52 days, p = 0.01). Similar findings have been noted by Otaka et al. [11] and Liu et al. [12], with patients who underwent surgical rib fixation having a shorter length of hospital stay (32.1 vs. 45.1 days; difference, 13.0 days; 95% CI: 1.3-24.6; p = 0.03) and (13.12 ± 4.21 days vs. 18.57 ± 5.39 days; p < 0.001), respectively.

In our study, the mean duration of ICU stay was significantly shorter among patients with operative management than with nonoperative management (2.72 ± 0.88 vs. 4.24 ± 0.01 days). Similar results have been reported by Bauer et al. [13] and Liu et al. [12], who also found that the mean duration of ICU stay was significantly shorter among patients undergoing SSRF than conservative management (8.72 ± 7.95 vs. 14.28 ± 9.06 days; p = 0.01) and (4.02 ± 1.41 vs. 5.06 ± 1.80 days; p = 0.001), respectively.

In the present study, the mean duration of mechanical ventilation was significantly shorter among patients undergoing operative management than among those undergoing nonoperative management (3.03 ± 1.15 vs. 5.38 ± 1.24 days; p = 0.01). In contrast to our findings, Bauer et al. [13] reported no difference in the mean duration of mechanical ventilation days between patients managed surgically and conservatively (8.22 ± 8.34 vs. 9.24 ± 6.5 days; p = 0.59). Fokin et al. [14] also found that the mean duration of mechanical ventilation was significantly longer among patients managed using SSRF than patients managed nonoperatively (3.2 vs. 1.6 days; p = 0.04).

Pain control and early mobilization are critical determinants of outcomes in patients with multiple rib fractures. SSRF has been increasingly recognized as an effective modality for reducing pain severity and improving pulmonary mechanics. In the present study, patients undergoing operative fixation demonstrated improved early recovery parameters, which can be attributed to the stabilization of the chest wall, reduction of paradoxical motion, and restoration of normal respiratory mechanics. Similar observations were reported by Marasco et al. [15], who demonstrated that surgical stabilization significantly reduced pain scores and improved pulmonary function compared with conservative treatment in patients with flail chest.

Another important observation in the present study was the reduction in pulmonary complications among surgically treated patients, particularly pneumonia and atelectasis, although this trend did not always reach statistical significance. This finding aligns with the results of Granetzny et al. [16], who reported a significant reduction in pulmonary infections in surgically managed patients compared to those treated conservatively. The stabilization of fractured ribs helps maintain adequate ventilation and reduces secretion retention, thereby lowering the risk of secondary respiratory infections.

Functional recovery and return to normal activity represent essential outcome parameters following rib fracture management. Patients treated surgically in the present study showed earlier ambulation and improved functional recovery compared with the nonoperative group. These findings are consistent with those of Pieracci et al. [8], who reported that SSRF was associated with improved functional outcomes, faster return to work, and better overall patient satisfaction. The improved stability of the thoracic cage allows patients to participate more effectively in physiotherapy and respiratory exercises, facilitating faster rehabilitation.

Another significant aspect observed in the present study was the reduced need for prolonged analgesia in the operative group. Adequate pain control remains one of the most challenging aspects of rib fracture management, and inadequate analgesia is associated with poor respiratory effort, hypoventilation, and increased risk of pulmonary complications. Qiu et al. [17], in a systematic review and meta-analysis, reported that SSRF significantly reduced narcotic usage and improved pain outcomes in patients with multiple rib fractures.

The good results obtained in the SSRF group may be attributed to the restoration of chest wall stability, improved respiratory mechanics, and better pain control that may facilitate early mobilization and reduce pulmonary complications. Previous studies have reported similar findings, showing decreased duration of ventilation, ICU stay, and hospital stay after SSRF.

Current recommendations from expert consensus groups and trauma societies suggest SSRF for patients with flail chest and selected patients with multiple displaced rib fractures, particularly in the presence of respiratory compromise, chest wall instability, or failure of conservative management. However, indications continue to evolve, and patient selection remains an area of active investigation.

Despite the favorable outcomes observed in operative management, it is important to acknowledge that SSRF is not without limitations. Surgical intervention requires specialized expertise, infrastructure, and financial resources, which may limit its widespread application, particularly in resource-constrained settings such as many trauma centers in developing countries. The financial constraints encountered in the present study also prevented patient randomization, which represents a methodological limitation. Similar challenges have been highlighted by Kasotakis et al. [18], who noted that economic and logistical barriers remain major obstacles to universal adoption of SSRF.

The quasi-experimental design of the present study represents another limitation that should be considered while interpreting the findings. Although baseline characteristics between the operative and nonoperative groups were comparable, the absence of randomization may introduce selection bias. Additionally, the sample size in the present study was relatively modest, which may limit the generalizability of the findings. Future studies with larger sample sizes and randomized controlled designs are required to validate these findings and further refine the indications for SSRF.

Another limitation relates to the duration of follow-up, as long-term complications such as chronic pain, chest wall deformity, and pulmonary dysfunction may manifest beyond the early postoperative period. Extended follow-up studies are necessary to better understand the sustained benefits of surgical stabilization over conservative management.

Despite these limitations, the findings of the present study contribute valuable data from a Level I Trauma Center in Eastern India, where evidence regarding SSRF remains limited. The results support the growing body of literature advocating early surgical stabilization in selected patients with multiple rib fractures, particularly those with flail chest or severe thoracic injury.

Formal cost-effectiveness analysis was not performed and represents an area for future research. Future research should focus on cost-effectiveness analysis of SSRF in low- and middle-income countries, optimization of patient selection criteria, and evaluation of minimally invasive fixation techniques. Also, standardized protocols for perioperative care, pain relief, and rehabilitation should be developed to maximize the benefits of surgical stabilization.

## Conclusions

The present quasi-experimental study compared SSRF with nonoperative management among patients aged 18-70 years. Both groups were demographically comparable in terms of age, sex, mode and side of injury, associated pulmonary complications, and comorbidities, with no statistically significant differences in these baseline characteristics. Key clinical outcomes favored operative management. Patients undergoing SSRF demonstrated significantly shorter durations of hospital stay, ICU stay, and ventilatory support. Additionally, significant improvements were observed in pain outcomes, as evidenced by lower VAS scores at follow-up, and in functional recovery.

Although financial constraints prevented randomization, the key clinical outcomes favored operative management. Patients undergoing SSRF demonstrated significantly shorter durations of hospital stay, ICU stay, and ventilatory support. Additionally, significant improvements were observed in pain outcomes, as evidenced by lower VAS scores at follow-ups, and in functional recovery, reflected by higher PIC.

Our study had limitations, as it was quasi-experimental and we could not randomize due to financial constraints. We had included a modest no of samples in the study. For the assessment of chronic pain and pulmonary function, longer follow-up is required.
